# Prominence and Expectation in Speech and Music Through the Lens of Pitch Processing

**DOI:** 10.3389/fpsyg.2021.620640

**Published:** 2021-07-08

**Authors:** Xiaoluan Liu

**Affiliations:** ^1^Department of English, East China Normal University, Shanghai, China; ^2^Department of Speech, Hearing and Phonetic Sciences, University College London, London, United Kingdom

**Keywords:** pitch prominence, melodic expectation, speech, music, perceptual processing

## Abstract

Speech and music reflect extraordinary aspects of human cognitive abilities. Pitch, as an important parameter in the auditory domain, has been the focus of previous research on the relations between speech and music. The present study continues this line of research by focusing on two aspects of pitch processing: pitch prominence and melodic expectation. Specifically, we examined the perceived boundary of prominence for focus/accent in speech and music, plus the comparison between the pitch expectation patterns of music and speech. Speech (Mandarin Chinese) and music stimuli were created with different interval steps that increased from 1 semitone to 12 semitones from the third to the fourth word/note of a sentence/melody. The results showed that ratings of both accent/focus and expectation/surprise increased with increasing semitone distance from the baseline (though this pattern was mixed with tonal stability profiles for the melodies). Nevertheless, the perceived boundary of prominence was different for music and speech, with the boundary for detecting prominence in speech higher than that in music. Expectation also showed different patterns for speech and music. The results thus favor the suggestion that speech prosody and music melody tend to require specialized pitch patterns unique to their own respective communication purposes.

## Introduction

Pitch change is an important source of information about our auditory environment, particularly in terms of speech and music. The rising and falling pitch patterns (i.e., melody) common to both speech and music have naturally given rise to the question as to what relations there may be between speech prosody and music melody (Bolinger, 1985). Currently, there are two major views regarding the relations between the two domains: One is that speech prosody and music melody processing share common cognitive resources although the surface representations of the two domains differ (Patel, 2008); the other is that the processing of speech prosody and music melody is largely separate (despite some similarities) due to differences in both surface structure and underlying neurophysiological mechanisms (Peretz, 2006, 2012; Zatorre and Baum, 2012). Evidence for each view mainly comes from studies on congenital amusia (cf. Peretz and Hyde, 2003; Patel, 2008), statistics of pitch patterning (Patel et al., 2006), and neuroimaging of normal and brain-impaired individuals (cf. Zatorre and Baum, 2012).

The present study is aimed at shedding new light on the above two views by exploring the relations between speech prosody and music melody from different perspectives: pitch prominence and expectation. They play a vital role in guiding the perceptual processing of melodic information in speech and music. This is because pitch prominence arises from sound events that are emphasized from the acoustic environment due to their prosodic salience (Terken and Hermes, 2000). Such prosodic salience usually helps direct listeners’ attention to acoustically important events, such as focus in speech or melodic accent in music, thus facilitating listeners’ comprehension of speech or music (Parncutt, 2003). With regard to expectation in the context of acoustic communication, it is a cognitive mechanism enabling listeners to anticipate future sound events (Meyer, 1956). It is one of the essential cognitive abilities for humans to adapt and survive because failure to predict and anticipate future events increases the risk of losing control and decreases the possibility of preparing for dangers (Huron, 2006). Violation of expectation, therefore, is likely to give rise to surprise (Reisenzein, 2000; Scherer et al., 2004). In this study, we will specifically concentrate on prosodic focus in speech (with Mandarin as the target language) and music melodic accent, as well as expectation patterns (i.e., the degree of surprise) in both speech prosody and music melody.

### Pitch Prominence in Speech and Music: Focus and Melodic Accent

**Figure 1:**
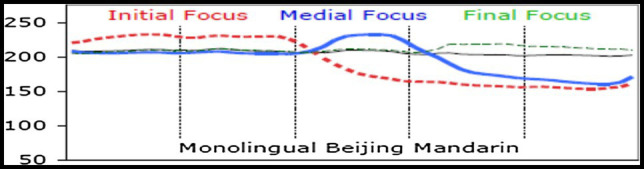
Time-normalized mean F0 contours produced by Mandarin speakers. The vertical lines represent syllable boundaries. The horizontal thin black line represents the no-focus condition, while the colored-lines represent focus conditions in different sentence positions (adapted from Xu, 2011).

In speech, focus is usually defined as highlighting the prominence of a piece of information in an utterance, thus facilitating listeners to differentiate the important from the unimportant in the speaker’s utterance (Rump and Collier, 1996). Focus could be materialized in different languages in different dimensions. This study is only concerned with the role of pitch in marking focus/accent, but it is worth pointing out that other acoustic features, such as duration and intensity, can also contribute to the perception of focus in speech. One of the essential ways of signaling focus in speech communication is by prosody (Cooper et al., 1985; de Jong, 2004), especially by pitch range expansion as has been evidenced from non-tonal languages (Liberman and Pierrehumbert, 1984) and tonal languages, such as Mandarin, where F0 height and pitch contour differences are used to contrast between lexical tones (Xu, 1999; Chen and Gussenhoven, 2008; see Figure 1 for schematic illustration of focus prosody in Mandarin). For example, Ouyang and Kaiser (2015) found that different types of focus in Mandarin (e.g., focus to signal correction and new information) were associated with pitch range variations, such as lengthening and expansion of the F0 range. Similarly, Chen and Gussenhoven (2008) investigated the F0 patterns of sentences with different degrees of emphasis (no emphasis, emphasis, and more emphasis) in Mandarin. The results showed that changing from no emphasis to emphasis condition involved a significant increase in F0 range, but changing from emphasis to more emphasis condition involved only marginal increase in F0 range, thus suggesting a non-gradual pattern of F0 range expansion for sentences with different degrees of emphasis. Tong et al. (2005) compared the processing of contrastive stress and sentence intonation in Mandarin. Their findings were in line with previous studies (e.g., Hickok and Poeppel, 2004) that the right hemisphere was primarily recruited in processing lower-level aspect of speech prosody, such as contrastive stress, but the left hemisphere was primarily involved in processing higher-level prosody, such as sentence intonation.

Nevertheless, with regard to the question of whether discrete pitch ranges exist for functions like focus, no consensus exists in the current literature. For example, Bruce (1977) and Horne (1988) have proposed specific target height of focused components for the sake of speech synthesis. Empirical studies have also provided psychological evidence. For instance, Rump and Collier (1996) have found that Dutch listeners tended to assign specific pitch values (ranging from 2 to 6 semitones higher than baseline) to focused syllables. Hart (1981) has found that differences of less than 3 semitones are not significant for the detection of large pitch movement in Dutch. Rietveld and Gussenhoven (1985) have found a smaller boundary of prominence, i.e., a pitch difference of 1.5 semitones was sufficient to enable listeners to perceive a difference in Dutch pitch prominence. On the other hand, controversial findings also exist as to the lack of discriminatory boundary for focus or accent (accent is also an acoustic measure of a prominent piece of information of an utterance; Lightfoot, 1970). For example, Ladd and Morton (1997) have found no discriminatory boundary between emphatic and non-emphatic accents in English. There have also been findings of lack of division of pitch range for different types of focus for Dutch (Hanssen et al., 2008) and English (Sityaev and House, 2003). The above interesting albeit somewhat controversial findings on the boundary of pitch prominence perception in non-tonal languages raise the question as to whether the same pattern could be found in tonal languages, such as Mandarin Chinese. So far, no empirical research has formally investigated this issue. Given the functional use of F0 for differentiating lexical words in Mandarin, it could be hypothesized that Mandarin speakers may not use the same pitch pattern as a cue for communicating focus to each other.

**Figure 2:**
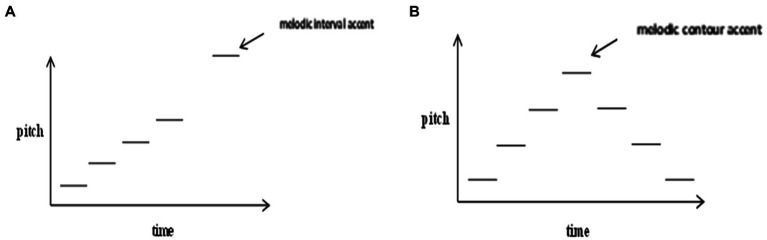
Melodic interval accent **(A)** and contour accent **(B)**.

In terms of music, accent is the counterpart of focus, as “(melodic) peaked contours might serve to highlight ostensively certain features of a musical utterance, a function analogous to that of focus in speech prosody” (Cross and Woodruff, 2009, p. 91). More specifically, similar to focus in speech, accents in music are noticeable sound prominences that deviate from contextual norms (Jones, 1987). One of the important ways of conveying accent in music is by pitch change, i.e., melodic accent which is often triggered by change in interval or contour and so is also called interval accent or contour accent (Huron and Royal, 1996). Interval accent most frequently occurs on the highest pitch after a large interval leap (Lerdahl and Jackendoff, 1983; Graybill, 1989; Figure 2A). The accent can be particularly prominent if the large interval leap is surrounded immediately by stepwise intervals (Graybill, 1989). Contour accent (Figure 2B) is proposed to occur at the pivot point where pitch direction changes (hence the pivot accent proposal), especially at the highest pitch of an ascending-descending contour (Thomassen, 1982). Huron and Royal (1996) using a large database with various music styles (e.g., British folk ballads and American popular melodies) showed strong support for the pivot accent proposal. Interval accent and contour accent often overlap since the highest pitch after a great interval leap often lies in the pivot position of the melodic contour (Hannon et al., 2004). The degree of melodic accent is proposed to be positively related to the size of pitch interval, i.e., the larger the interval size, the stronger the degree of accent (Lerdahl and Jackendoff, 1983). Nevertheless, so far it is not clear as to how large the interval size should at least be to evoke the perception of melodic accent.

### Expectation in Speech and Music

Expectation is part of psychological laws of mental life responsible for human perception and cognition (Meyer, 1956). More specifically, it is a cognitive mechanism enabling humans to make predictions about the development of future events (Meyer, 1956). Expectation is often reflected in the extent of surprise: A low degree of surprise can reflect consistence with expectation, while a high degree can reflect violation of expectation (Scherer et al., 2004). In particular, surprise in this study refers to the listener’s surprise upon hearing musical patterns that are novel and inconsistent with previous listening.

In speech, surprise also refers to the violation of previously maintained expectation for ongoing speech. With regard to prosody, the intonation of surprise is characterized by a large pitch range expansion and a relatively high pitch level (Gussenhoven and Rietvelt, 2000; Lai, 2009). In Mandarin, surprise is associated with high mean F0 and large F0 variation, as evidenced from a large database of Mandarin vocal emotional stimuli (Liu and Pell, 2012). Absence of such prosodic cues, e.g., compression or flattening of the pitch contour, could lead to an indication of no surprise or information withdrawal (Gussenhoven, 2004; Lai, 2009). The prosodic characteristics of focus and surprise are closely linked, for the reason that prosodically prominent speech elements, such as focus and stress, are often the main carriers for signaling surprise, as has been evidenced from German (Seppi et al., 2010).

In music, the degree of surprise is often triggered by different melodic expectation patterns, which have been theorized by Narmour (1990, 1992) in his influential implication-realization (I-R) model of melody. Following Meyer (1956) and Narmour (1990) used “implication” to refer to melodies generating expectations and “realization” as melodies fulfilling expectations. The core idea is that melody perception is built on melodic implications which arise from listeners’ expectations for the following melodic events triggered by the preceding events. The events particularly refer to musical intervals. The principles of the I-R model have been summarized into five key principles for melodic expectation (cf. Krumhansl, 1995a,b). Of particular relevance to this study is the proximity principle, i.e., smaller intervals are generally more expected than large intervals (Narmour, 1990). This is based on the observation that small intervals tend to be predominant in various music styles (Meyer, 1973; Narmour, 1990). Vos and Troost (1989), for example, used synthetic musical stimuli to test the perceptual relevance of the distributional regularity of melodic intervals in Western music. Their findings were consistent with the claim that larger intervals often trigger a sense of discontinuity in melody, which as a consequence tends to disrupt a listener’s expectation for the progression of a melodic pattern (Meyer, 1973). Consequently, a number of studies have used perception and production methods to test the principles of the I-R model. The results on the one hand largely supported the model while on the other hand found the need to include additional factors of tonality (e.g., tonal strength, consonance, tonal stability, and tonal hierarchy) to boost the model’s predictive power (Cuddy and Lunney, 1995; Krumhansl, 1995a,b; Thompson et al., 1997). The reason is that musical elements (e.g., tones, chords, and keys) are often linked to one another. Such close links reflect ‘the connection between melodic and harmonic organization and between the musical elements actually sounded and a system of interrelated key regions (Krumhansl, 1983, p. 59).’

The I-R model also has the potential to explain the intonation patterns in speech, as once tentatively outlined in Narmour (1991). This is because the I-R model is built on the idea that human’s expectation patterns are governed by principles that can be applied universally (Narmour, 1990). The principles of the model, therefore, are relevant to all types of melody/prosody (e.g., music or speech; Narmour, 1991). Indeed, the above review on the pitch patterns of surprise in speech and music suggests that in both domains, small intervals (i.e., small pitch excursions) are generally less likely to trigger surprise than large intervals. The reason could be explained by common motor and perceptual constraints (Patel, 2008). This could serve as further evidence for the close link between speech and music with regard to expectation (Patel, 2008). It is worth pointing out that although pitch in speech does not strictly follow frequency ratios (i.e., semitone intervals) in the same way as music does, research has shown that pitch intervals may indeed be essential to the perception of speech intonation (Hermes, 2006). Evidence can be found in neutral speech (Patel et al., 2006), emotional speech (Curtis and Bharucha, 2010), and stylized interjections (Day-O’Connell, 2013). Moreover, pitch intervals were adopted as a paradigm for examining pitch perception in speech a long time ago (Rietveld and Gussenhoven, 1985; Rump and Collier, 1996). In addition, the use of semitone intervals facilitates cross-modal comparisons between speech and music in terms of pitch processing. Therefore, it is worth testing Narmour’s (1991) argument by empirically examining whether in a tonal (and hence melodic) language, like Mandarin, principles of the I-R model can be truly applicable in the same way as they are to music.

### The Present Study

The above review suggests that firstly, both speech focus and music melodic accent are mediated by pitch prominence, but there is not a clearly established boundary of prominence for the perception of focus in Mandarin and melodic accent in music. Also, it is not known whether and how music and speech differ in the boundary of pitch prominence. Secondly, it would be interesting to test whether speech and music follow the same principles of the I-R model in terms of expectation violation. Although plenty of previous studies have investigated the relations between speech prosody and music melody, so far there is little research on whether or not speech and music follow the same pitch patterns in signaling prominence and expectation. A proper understanding of this question will contribute to the theoretical debate about the extent to which pitch processing mechanisms are shared between speech and music (Patel, 2008; Peretz, 2012). Some studies have shown that the two domains are closely connected. For example, Hausen et al. (2013) investigated how music perception was related to speech prosody perception using different types of tasks (scale, rhythm, and word stress tests), and found a robust link between the two domains. Morrill et al. (2015) investigated the relations between music and speech prosody processing by controlling for individual differences in cognitive ability. Their finding supported a domain-general account of a shared mechanism between music and speech with respect to pitch processing. Angenstein et al. (2012) directly compared the processing of pitch intervals in music and speech by using sequences of the same spoken or sung syllables, and they found that both bottom-up and top-down (i.e., speech mode, pitch interval, and task) effects could influence the listeners’ processing of the pitch intervals. Patel et al. (1998) tested amusic listeners’ ability to process melodic and rhythmic patterns in speech and music. The results suggested cross-domain similarity between speech and music, thus leading to the possibility that prosody and music may share neural resources. Similarly, Schön et al. (2004b) used behavioral and neurophysiological methods to investigate the time course of pitch processing in speech and music by musicians and non-musicians. The results showed that F0 manipulations of both music and language stimuli triggered similar brain activity patterns, suggesting a shared mechanism of pitch processing between music and language. Nevertheless, some studies have also found that there could be some discrepancies between music and speech in pitch processing. For example, brain lesion studies have found that patients with language impairments can still maintain their abilities to sing after losing their ability to speak (Peretz et al., 2004; Wilson et al., 2006). Conversely, singing can be impaired exclusively (Peretz, 2012). For example, Schön et al. (2004a) reported that an opera singer who had lost the ability to sing intervals could still produce correct speech intonation. Similarly, Ayotte et al. (2002) reported cases of amusic adults who could not sing accurately but could still speak normally. Saito et al. (2006) identified a neural network (the right inferior frontal gyrus, the right pre-motor cortex, and the right anterior insula) in singing that was snot shared in speaking. Therefore, the above findings lead to the suggestion that music and language could be processed in a domain-specific fashion (Peretz, 2012). Specifically, the processing of speech prosody and music melody could be largely separate (despite some similarities) due to differences in both surface structure and underlying neurophysiological mechanisms (Peretz, 2006, 2012; Zatorre and Baum, 2012).

The above suggests that there could be an intriguing relation between speech and music in terms of pitch processing. Nevertheless, some fundamental issues have not been investigated properly, especially with regard to pitch prominence and expectation patterns in speech and music. Hence, this study explores the following research questions: (1) What are the boundaries of prominence for the perception of focus in speech (Mandarin) and melodic accent in music? Are music and speech different in the boundary of pitch prominence? (2) Is the I-R models’ proximity principle applicable to speech (Mandarin) in the same way as it is to music in terms of expectation violation? It is possible that music and speech will have their specific boundaries of prominence, and the I-R model could apply to both music and speech, but due to the tonality constraints unique to music melody (as discussed in section “Expectation in Speech and Music”), the exact boundary of triggering expectation violation (i.e., surprise) may differ between music and speech.

## Speech and Music Experiments

The experiments were designed to address research question 1 (focus/accent) and question 2 (expectation/surprise) with the same experimental materials. This is because, in speech, prosodically prominent elements, such as focus, are often the main carriers for signaling surprise (Seppi et al., 2010); similarly in music, melodic accents often function to signal musical surprise as well (Jones, 1987). Hence, by making one component in either speech or music prosodically prominent, two research questions (focus/accent and surprise) can be tackled at the same time. Also note that for research question 2, this study only explores the condition where pitch direction remains unchanged, because surprise in speech usually involves continuous pitch expansion in the same pitch direction rather than the other way round (cf. Kreiman and Sidtis, 2011).

### Methods

The study was approved by the UCL Research Ethical Committee. All experiments were performed according to relevant guidelines and regulations.

#### Participants

Two groups of participants were recruited: 15 native Mandarin speakers with professional musical training background (average training time = 20 years, 9 females, age *M* = 31 years, *SD* = 3.6) and another group of 15 Mandarin speakers without musical training background (7 females, age *M* = 28, *SD* = 2.2). They reported no speech or hearing problems.

#### Stimuli

##### Speech

**Figure 3:**
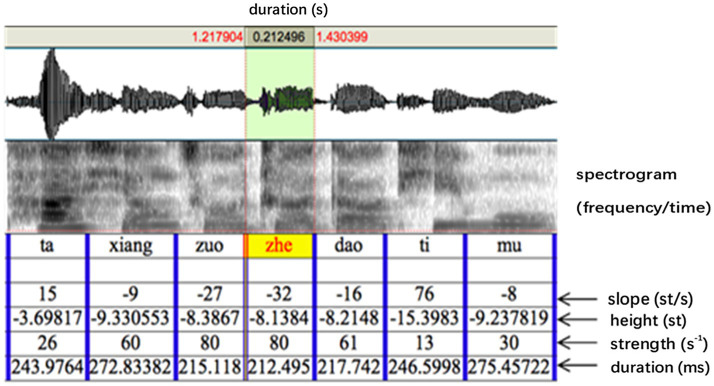
The segmentation of the stimulus sentence (“zhe” as the target syllable) with parameters automatically derived from PENTAtrainer1 through analysis by synthesis (Xu and Prom-on, 2010).

**Figure 4:**
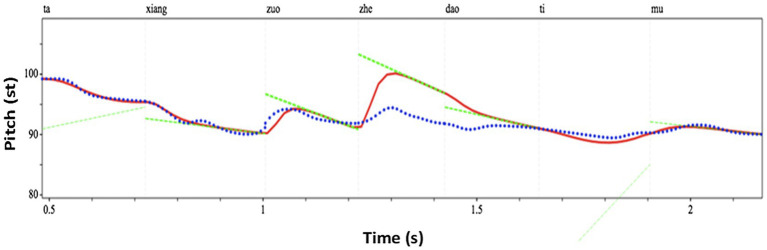
An example (6 semitones above the baseline of “zhe”) of the synthesized speech stimuli using PENTAtrainer 1 (Xu and Prom-on, 2010). The blue line represents the original speech contour. The red line represents the synthesized speech contour. The green line represents the pitch target parameters.

A pre-recorded sentence “Ta (tone1) xiang (tone3) zuo (tone4) zhe (tone4) dao (tone4) ti (tone2) mu (tone4)” (He wanted to solve this problem) spoken in a neutral way (i.e., without focus on any syllable) by a native Mandarin Chinese speaker was used as the base sentence. PENTAtrainer1 (Xu and Prom-on, 2010) running under Praat (Boersma and Weenink, 2013) was used to synthetically modify the F0 contours of the sentence (similar to PSOLA) in such a way that the prosody sounds natural despite the large pitch range modifications. PENTAtrainer1 was based on the PENTA model (Parallel Encoding and Target Approximation) proposed in Xu (2005). The PENTAtrainer1 script was developed from the qTA (quantitative target approximation) implementation (Prom-on et al., 2009) of the PETNA model. The rationale of the model is that pitch contours of tone and intonation can be simulated as a result of syllable-synchronized target approximation, under the assumption that speech production functions under both biomechanical and linguistic mechanisms (Prom-on et al., 2009). More specifically, the program first extracts for each (manually segmented) syllable an optimal pitch target defined for its height, slope, and strength. It then allows the user to arbitrarily modify any of the target parameters and then resynthesize the sentence with the artificial target. Figure 3 shows the segmented syllables with the parameters extracted by PENTAtrainer1. For experiment 1, the syllable “zhe” (this) was used as the target syllable. Its pitch height parameter (as shown in Figures 3, 4) was incrementally raised up to 12 semitones (in one-semitone steps) according to the pitch height of the pre-focused syllable (zuo; more explanation of this is offered below): b = − 8.1384 (the pitch height of zuo) + 1 (semitone), + 2 (semitones), + 3 (semitones)…+ 12 (semitones). One semitone was chosen as the step size because a pilot study showed that listeners could not significantly distinguish pitch differences of less than one semitone.

Note that in this study, the pre-focused (zuo), focused (zhe), and post-focused (dao) syllables all have the same falling tone (Tone 4) in Mandarin. Therefore, the pitch manipulation of the focused syllable with reference to the pitch of the pre-focused syllable (as was done in this study) is similar to the pitch manipulation of the focused syllable with reference to the pitch of the post-focused syllable. Such design allows the comparison of this study with previous studies on speech focus while enabling the comparison of speech with music in pitch prominence and expectation: Previous studies on focus perception (in non-tonal languages) manipulated the pitch of focus according to the baseline (i.e., neutral) condition of the focused syllable itself rather than the pre-focused syllable as in this study. While, in this study, speech has to be manipulated in the same way as music (the details are provided in the following section) in order to facilitate comparison between them. This means the component (speech syllable or musical note) should be manipulated according to the pitch of the component immediately preceding the manipulated one (because this is how melodic accent and expectation function in music). Therefore, by making the pre-, on-, and post-focused syllables share the same tone (tone 4), we can guarantee that any of them can serve as the reference (baseline), thus enabling comparisons within this study (speech and music) and across studies (this study and previous studies on speech focus; cf. Prom-on et al., 2009; for technical details of the extraction of pitch by PENTAtrainer1).

It is also worth mentioning the reason for selecting tone 4 for manipulation is that it produces the clearest pitch target manipulation contour under PENTAtrainer 1 according to our pilot studies. Moreover, the pilot studies showed that listeners’ judgment patterns did not differ significantly between stimuli manipulated based on tone 4 and stimuli manipulated based on the rest of the tones (tones 1, 2, and 3).

##### Music

**Figure 5:**
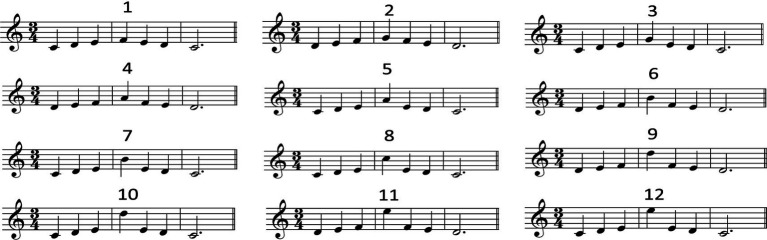
The 12 short excerpts composed as the music stimuli. Each excerpt corresponds to a different interval size between the third and fourth note (1 = 1 semitone, 2 = 2 semitones … 12 = 12 semitones).

Twelve short excerpts in C major were composed for this study (Figure 5). Similar to speech, the fourth component (musical note) was the target of manipulation: Its pitch height ranged from one semitone above its preceding note all the way to 12 semitones above. Therefore, the target components (syllable or note) in speech and music followed the same manipulation patterns of pitch increase relative to their respective preceding components. This design enables the comparison between speech and music in terms of pitch prominence and expectation.

Note that two different starting tones were used for the melody composition, e.g., do re mi fa mi re do (the first panel of Figure 5) and re mi fa so fa mi re (the second panel of Figure 5). The reason is that if we stick to one starting tone (e.g., do), then inevitably some of the manipulated notes will be chromatic (i.e., mainly the black keys in the context of C major), for example, under the condition where the target note is 2 semitones above its preceding note (e.g., E–#F). Chromatic tones within C major are highly dissonant and unpleasant (Krumhansl, 1990) and hence would have an impact on listeners’ response in terms of melodic expectation. Therefore, in this study two starting tones were used for the stimuli composition to avoid the possible occurrence of chromatic tones.

Each note of the melody was of equal amplitude (56 dB) and was 0.5 s in duration except the last note (which was three times as long as the previous note because it was a dotted half note in time signature 3/4). This was so designed as to avoid the possible contribution of intensity and duration to the perception of prominence (accent; Ellis and Jones, 2009), since the focus of this study was on melodic (pitch) prominence. The total duration of each melody was 4.5 s. All melodies were created using Finale 2011 (piano sound).

#### Procedure

For the speech experiment, the stimulus sentence was presented three times in a pseudorandom order on a computer. Listeners performed two tasks on separate days: For the first task, they rated the degree of focus conveyed by the syllable “zhe” (this) of every sentence on a scale of 1–3 (1= no focus; 2 = focus; and 3 = a strong degree of focus). Then, a week later, they were invited back to finish the second task. The stimuli for the second task were the same as the first task, but listeners were asked to rate the degree of surprise conveyed by the syllable “zhe” of each sentence on a scale of 1–3 (1= not surprising; 2 = surprising; and 3 = very surprising). In particular, surprise means the participants’ surprise after hearing the stimuli. To insure listeners can distinguish between “focus” and “surprise,” different pragmatic contexts were provided. For focus, the context was: He wanted to solve this rather than that problem. For surprise, the context was: It was so surprising that he (a very clever student) wanted to solve this problem in an intelligence contest. The problem was so simple that even a not-so-clever student could easily solve, and it turned out that he (with superb intelligence) wanted to solve this problem to show how clever he was.

The music experiment was carried out on a different day than the speech experiment. Similar to the speech experiment, each melody was presented three times in a pseudorandom order on a computer. The same group of listeners participated in the experiment and performed two tasks: For the first task, they rated the degree of melodic accent conveyed by the fourth note of every melody on a scale of 1–3 (1= no melodic accent; 2 = melodic accent; and 3 = a strong degree of melodic accent). The participants were briefed before the tasks what melodic accent refers to and they were given a practice section (with stimuli different from the experimental task) to familiarize themselves with this concept. A week later, they were invited back to finish the second task. The stimuli for the second task were the same as the first task, but listeners were asked to rate the degree of surprise (i.e., how out of expectation when listening to the string of notes) conveyed by the fourth note of each melody on a scale of 1–3 (1= not surprising; 2 = surprising; and 3 = very surprising).

For all the experimental sessions, counterbalancing of the tasks and experiments was used to minimize the order effect.

### Results

Mixed ANOVAs with a between-subject factor (group: musicians vs. non-musicians) and two within-subject factors (type: music vs. speech; interval size) were conducted for the conditions of prominence (focus in speech and melodic accent in music) and surprise, respectively. The results showed that the main effect of group was non-significant in both the prominence and surprise conditions, i.e., no significant differences were found between musicians and non-musicians in terms of their ratings of pitch prominence [*F*(1, 28) = 0.51, *p* = 0.48] or surprise [*F*(1, 28) = 0.02, *p* = 0.89]. Further, no significant interactions were found between group and other factors (type, interval) in the prominence condition: group * type [*F*(1, 28) = 0.053, *p* = 0.82], group * interval [*F*(11, 308) = 0.12, *p* = 0.998], and group * type * interval [*F*(11, 308) = 0.09, *p* = 0.99], or in the surprise condition: group * type [*F*(1, 28) = 0.22, *p* = 0.65], group * interval [*F*(11, 308) = 0.11, *p* = 0.99], and group * type * interval [*F*(11, 308) = 0.14, *p* = 0.99].

Furthermore, the main effect of type was significant in both conditions; i.e., speech and music were significantly different in terms of prominence [*F*(1, 28) = 24.4, *p* < 0.001, $\eta_{p}^{2}$ = 0.47] and surprise [*F*(1, 28) = 34.18, *p* < 0.001, $\eta_{p}^{2}$ = 0.55]. Meanwhile, the main effect of interval size was significant in both conditions as well; i.e., different interval sizes corresponded to significantly different ratings of prominence [*F*(11, 308) = 194.14, *p* < 0.001, $\eta_{p}^{2}$ = 0.87] and surprise [*F*(11, 308) = 224.59, *p* < 0.001, $\eta_{p}^{2}$ = 0.9]. More details are provided below.

#### Speech

**Figure 6:**
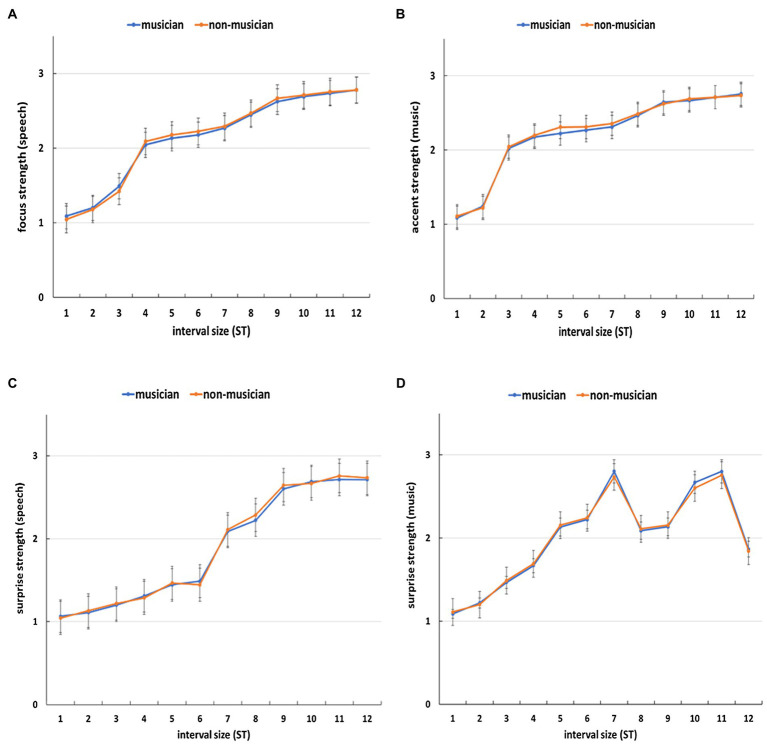
The average ratings of focus/accent strength [**(A)** for speech and **(B)** for music] and surprise strength [**(C)** for speech and **(D)** for music] for each interval size (ST = semitone). Error bars represent standard error of the mean.

The results showed that the larger the interval size, the higher the ratings of the strength of focus (Figure 6A) and surprise (Figure 6C). This is further confirmed in a one-way repeated measures ANOVA [focus: *F*(11, 319) = 125.4, *p* < 0.001, $\eta_{p}^{2}$ = 0.81; surprise: *F*(11, 319) = 226.2, *p* < 0.001, $\eta_{p}^{2}$ = 0.89], where interval size had a significant main effect on the strength of focus and surprise, respectively. Furthermore, for focus from 4 semitones onward (Figure 6A) and for surprise from 7 semitones onward (Figure 6C), the average ratings for focus strength and surprise strength, respectively, were above two which is the boundary between no focus/not-surprising (i.e., the rating of 1) and focused/surprising (i.e., the rating of 2). A one-way repeated measures ANOVA further showed that for focus, the difference in ratings between 3 semitones and 4 semitones was significant [*F* (1, 29) = 80.85, *p* < 0.001, $\eta_{p}^{2}$ = 0.74], while for surprise, the difference in ratings between 6 semitones and 7 semitones was significant [*F*(1, 29) = 55.39, *p* = 0.003, $\eta_{p}^{2}$ = 0.66]. This suggests an interval of at least 4 semitones was needed for the perception of focus and that of 7 semitones for the perception of surprise.

#### Music

For melodic accent, Figure 6B shows that the larger the interval size, the higher the rating of accent. This is further confirmed in a one-way repeated measures ANOVA [*F* (11, 319) = 107.7, *p* < 0.001, $\eta_{p}^{2}$ = 0.79], where interval size had a significant impact on accent strength. Moreover, Figure 6B shows from 3 semitones onward, the average ratings were above two (the boundary between no accent =1 and accent =2) and the difference in ratings between 2 semitones and 3 semitones was significant [*F* (1, 29) =184.24, *p* < 0.001, $\eta_{p}^{2}$ = 0.86]. This indicates that an interval of at least 3 semitones was needed for the perception of melodic accent. With regard to surprise, the results again showed a significant main effect of interval size on surprise strength [*F*(11, 319) = 113.7, *p* < 0.001, $\eta_{p}^{2}$ = 0.8]. Nevertheless, Figure 6D shows that only a partial relation existed: In the range of 1–7 semitones, the bigger the interval size, the higher the surprise strength and this was especially true from 5 semitones onward, where the average rating was above two (the difference between 4 and 5 semitones was significant [*F*(1, 29) = 67.67, *p* < 0.001, $\eta_{p}^{2}$ = 0.7]. However, after 7 semitones, the patterns of surprise strength became more irregular. The surprise strength of 8 semitones was lower than that of 7 semitones and the largest interval (12 semitones) did not correspond to the highest rating of surprise.

## Discussion

### Pitch Prominence in Speech and Music

In terms of pitch prominence (focus) in speech, the results of the experiments showed that the strength of focus increased as the pitch excursion size increased, with the boundary lying at 4 semitones, i.e., a pitch excursion of at least 4 semitones was needed to evoke listeners’ perception of focus in Mandarin. The results are consistent with previous findings that focus in Mandarin is associated with an increase in F0 value and range (Chen and Gussenhoven, 2008; Ouyang and Kaiser, 2015). Moreover, the results suggest that different from English or Dutch where the existence of boundary of focus is questionable (Sityaev and House, 2003; Hanssen et al., 2008), in Mandarin there could exist a discriminatory boundary of prominence for focus, which lies at 4 semitones above the base line. This further suggests that Mandarin speakers may not use the same pitch pattern to communicate focus as non-tonal language speakers, probably due to the functional use of F0 for differentiating lexical words in Mandarin (Xu, 1999).

With regard to pitch prominence (melodic accent) in music, the results showed that a pitch increase of 3 semitones was needed to convey melodic accent. As the interval size increased, the perceived strength of melodic accent also increased. The strongest degree of melodic accent appeared at the largest interval leap, i.e., 12 semitones in this study. The results are thus consistent with theoretic proposals that interval size in music is positively correlated with accent strength, especially in the context of large interval leap (Lerdahl and Jackendoff, 1983; Monahan et al., 1987; Drake et al., 1991).

The results suggest that speech and music are both similar and different in conveying pitch prominence. They are similar because in both domains, high pitch corresponded to a high degree of prominence. This is consistent with previous observation that pitch height is a marker of prosodic prominence in acoustic communications, such as speech and music (Parncutt, 2003; Patel, 2008). An acoustic dimension (such as pitch) with high salience usually attracts greater perceptual weight than that with low salience (Hart et al., 1990; Benward and White, 1997). Nevertheless, the results also showed difference in boundaries for pitch prominence: The boundary of speech focus was one semitone higher than that of music melodic accent. The reason could be that pitch is a fundamental building block in music (Patel, 2008) while in speech less so. This is evidenced from the finding that removing pitch information (i.e., F0) in speech does not inevitably harm intelligibility, even in a tonal language like Mandarin (Patel et al., 2010). A slight alteration of pitch in music, on the other hand, can easily be heard as “out of tune,” a concept that does not apply to speech (Zatorre and Baum, 2012). Therefore, a small change in pitch in music can lead to a significant change in musical meaning (such as melodic accent), whereas in speech, the magnitude of change in pitch does not need to be as subtle as that in music, even in tonal languages, such as Mandarin as shown in this study. Indeed, as argued in Peretz and Hyde (2003), linguistic prosodic contours are often less subtle than music melodic contours; i.e., music has a more fine-grained requirement for pitch compared with speech. Therefore, in music, the functional boundary (such as that of pitch prominence) needs to be lower (and hence more subtle) than that in speech, as demonstrated in the present study.

In summary, for research question 1, the results of this study showed that in both speech and music, high pitch generally corresponded to a high degree of prominence. Nevertheless, pitch perception boundary for focus in speech (Mandarin) was one semitone higher than that for the melodic accent in music. The differences between speech and music shown in the two experiments were due to the different functional requirements for pitch in speech and music.

### Expectation in Speech and Music

The results of the experiments showed that in both speech and music, small intervals were associated with low degree of expectation violation (i.e., surprise). This is consistent with the I-R models’ proximity principle, especially in terms of music: Smaller intervals are generally preferred over large intervals to avoid violation of expectation. The results on music were compatible with the principle because the degree of surprise was very low until the interval of 5 semitones, after which the degree of surprise became significantly large. With regard to speech, the results were in the same direction as predicated by the I-R model; i.e., small interval continuation corresponded to low level of surprise. This pattern is also consistent with previous studies, where a large pitch range expansion and a high pitch level are needed to trigger a sense of surprise in speech (Gussenhoven and Rietvelt, 2000; Lai, 2009; Liu and Pell, 2012), while a compressed pitch range usually indicates no surprise (Gussenhoven, 2004). Such preference for small intervals can be associated with our language experience (Patel, 2008). This is because greater frequency differences in vocal communication often correspond to larger intervals between pitch targets. According to Fitts (1954) law, muscular movement is more accurate between short-distance targets (e.g., small pitch intervals) than long-distance targets (e.g., large pitch intervals). Therefore, vocal communication in large frequency difference can be less accurate than that in small frequency difference and is thus less economical in speech articulation. Hence, it is the principle of economy of communication (in speech and music) that leads to the shared preference for small intervals in both domains, and the principle itself could be the results of common motor and perceptual constraints (Patel, 2008).

On the other hand, although speech was consistent with the direction of the I-R model’s prediction, the exact boundary for expectation violation (i.e., surprise) did not fall into the predicted range: In this study, the interval difference between “xiang” and “zuo” (the interval preceding the manipulated interval) was around 1 semitone, and according to the principle, the following interval should be within the range of 1 + 3 = 4 semitones in order not to trigger a large extent of surprise. Nevertheless, the results on speech showed that it was from 7 semitones onward that a large degree of surprise was triggered. Therefore, the results suggest a higher boundary for speech surprise perception than predicted by the I-R model. Moreover, speech had a higher boundary (7 semitones) for violation of expectation than music (5 semitones). The reason for such results is probably that in tonal languages, such as Mandarin, pitch serves to differentiate lexical items. Hence, there needs to be enough space for pitch to realize its function as a lexical marker. Consequently, paralinguistic meanings, such as surprise, have to be allocated to the remaining pitch space. Given the fact that in speech communication pitch range variation for linguistic information is usually kept small due to the need for economy of articulation (cf. Patel, 2008), the remaining large range of pitch variation is thus allocated to conveying paralinguistic meanings, such as surprise. This is also consistent with the findings that surprise intonation usually involves a large pitch excursion and high pitch level (Gussenhoven and Rietvelt, 2000; Lai, 2009). Meanwhile, such inconsistency with the I-R model’s prediction also supports the argument that unlike music, speech does not need to strictly follow interval ratios to communicate meaning (Zatorre and Baum, 2012).

In terms of large intervals, speech and music showed significant differences. The results demonstrated that large intervals in speech generally corresponded to a large extent of surprise (which was consistent with the I-R model), whereas in music, there was not a direct relation between interval size and the degree of surprise in the range of large intervals (from 8 semitones onward). For example, the interval of 8 semitones had a weaker degree of surprise than 7 semitones; the interval of 12 semitones was weaker in surprise than the intervals of 10 and 11 semitones. The reason for this could be associated with the influence of additional factors, such as tonal stability. More specifically, previous studies (Krumhansl, 1995b; Thompson et al., 1997) have reported that tonally less stable notes are generally perceived as more surprising than tonally stable notes. In this study, the 7-semitone interval ended in ti (the leading note) which is the least stable note in C major due to its inclination to resolve to the tonic do. This could lead to a high degree of surprise. In contrast, the 8-semitone interval ended in do, which is the tonic of the musical key it is situated in (C major). It is the most stable note (Meyer, 1956) and is therefore less surprising than the leading note. The 12-semitone interval, despite being the largest interval, was rated less surprising than smaller intervals (e.g., 10 and 11 semitones). The reason is that it ended in mi which is the median of C major (the musical key it is situated in). Since the median is the third most stable note of a musical key (after the tonic and the dominant, cf. Meyer, 1956), it is consequently less surprising, especially when compared with intervals of 10 and 11 semitones (the minor and major seventh) which require resolution to the tonic and hence less stable (Meyer, 1956). Such tonal stability exists only in music rather than in speech, and therefore, in the present study, pith expectation patterns of music were different from those of speech.

It is worth noting that the way the melodic stimuli were constructed in the present study could lead to possible confounds: The stimuli were designed to avoid chromatic tones because they could introduce dissonance and trigger unpleasant response among listeners, but this could lead to the possibility that the observed effects of interval size on perception may instead be due to tonal function or pitch height. More specifically, the notes in the melodies were limited to the C major diatonic scale and thus were likely to establish a C major tonality over the course of the experiment. This could mean that each pitch distance is associated with a different tonal function (e.g., the 2-semitone distance always occurred with the Dominant tonal function). The invariant absolute pitch height (e.g., the 2-semitone distance is always F4–G4) means that absolute pitch height could also be confounded with interval size. Although these confounds do not completely invalidate the study, they suggest that certain aspects of the results may be effects of pitch height or tonal function, rather than interval size *per se*.

The above findings suggest that the differences between music and speech outweighed the similarities between the two domains due to functional differences of pitch in speech and music. This is consistent with previous studies where some discrepancies were found between music and speech in terms of pitch processing (Ayotte et al., 2002; Peretz et al., 2004; Wilson et al., 2006). Although a direct comparison of the present study with previous research is not easy due to differences in research questions and design, the present study does lend support to the proposal that despite partial overlap, speech and music tend to be processed and produced in domain-specific ways because of differences in both surface structure and underlying neurophysiological mechanisms (Peretz, 2012; Zatorre and Baum, 2012).

In summary, for research question 2, the results suggest that in terms of small intervals, speech (Mandarin) and music were similar in the sense that both were consistent with the prediction of the I-R model: Small intervals were preferred over large intervals to avoid expectation violation (e.g., surprise). Nevertheless, the model could not predict the exact pitch boundary for surprise in speech (which was higher than music). In addition, in terms of large intervals, music was noticeably different from speech due to tonal constraints in music, such as pitch height or tonal function, which have no counterpart in speech.

## Conclusion

In conclusion, this study compared speech and music from two fundamental aspects: pitch prominence (i.e., focus in speech and melodic accent in music) and melodic expectation (i.e., the degree of surprise) within the framework of the I-R model. The results suggest that there can be some extent of overlap between speech and music in terms of pitch prominence (e.g., high pitch corresponded to great prominence) and expectation patterns (e.g., small intervals were preferred over large intervals). Nevertheless, the differences seemed to have outweighed the similarities between the two domains due to functional differences of pitch in speech and music. Therefore, in terms of the two views regarding the relations between speech prosody and music melody as introduced in section “Introduction,” the results are more in favor of the second view: Speech prosody and music melody tend to require specialized pitch patterns unique to their own respective communication purposes (Peretz, 2006, 2012; Zatorre and Baum, 2012). Hence, through the lens of pitch which is a fundamental parameter in the auditory domain, this study contributes to the disentanglement of the connections between speech and music from two fresh perspectives: pitch prominence and melodic expectation. Future studies could further investigate the possible interactions between interval size, pitch height, and tonal function in music and speech to advance our understanding of the intricate relations between the two domains.

## Data Availability Statement

The raw data supporting the conclusions of this article will be made available by the author, without undue reservation.

## Ethics Statement

The studies involving human participants were reviewed and approved by the Ethics Committee of University College London. The patients/participants provided their written informed consent to participate in this study.

## Author Contributions

XL designed the study, analyzed the data, and wrote the paper.

### Conflict of Interest

The author declares that the research was conducted in the absence of any commercial or financial relationships that could be construed as a potential conflict of interest.
